# Perceived Impact of Cutaneous Hyperpigmentation After Venous Procedures on Patient Self-Esteem: An Observational, Retrospective, Descriptive, Single-Center Case Series

**DOI:** 10.7759/cureus.116504

**Published:** 2026-09-18

**Authors:** Brenno Augusto S Mello Netto, Eduardo Zeilmann, Gilmar S Santos, Lucas Sarcinelli Alencar, João Pedro Hemerly, Vinicius Berro Natal, Diana De Oliveira Frauches

**Affiliations:** 1 Vascular Surgery, Seabra Excelencia Vascular, Vitória, BRA; 2 Vascular Surgery, Instituto de Flebologia Moderna, Itajaí, BRA; 3 Vascular Surgery, Vein Clinic, Salvador, BRA; 4 Vascular Surgery, Escola Superior de Ciências da Santa Casa de Misericórdia de Vitória, Vitória, BRA; 5 Medicine, Escola Superior de Ciências da Santa Casa de Misericórdia de Vitória, Vitória, BRA; 6 Surgery, Escola Superior de Ciências da Santa Casa de Misericórdia de Vitória, Vitória, BRA

**Keywords:** body image, case series, hyperpigmentation, patient-reported outcome measures, post-sclerotherapy hyperpigmentation, sclerotherapy, self-esteem, varicose veins, venous disease

## Abstract

Background and objective: Cutaneous hyperpigmentation following venous procedures is commonly regarded as an aesthetic complication of limited clinical relevance. Its effect on patients’ self-esteem and aesthetic perception, however, remains underexplored in the medical literature. This study aimed to describe the perceived impact of hyperpigmentation on self-esteem among patients who developed cutaneous hyperpigmentation after venous procedures.

Materials and methods: This was a retrospective, descriptive, single-center case series including a total of 91 consecutive patients with cutaneous hyperpigmentation following venous procedures who were treated between 2019 and 2025 at a specialized private vascular clinic. Data were extracted from a structured, non-validated questionnaire routinely applied in clinical practice, covering sociodemographic and clinical variables, aesthetic perception, and a self-reported rating of the perceived impact of hyperpigmentation on self-esteem (none, mild, moderate, or high). Variables were summarized using absolute and relative frequencies.

Results: Of the 91 patients included in the study, most were female (n=85, 93.4%), aged 30-49 years (n=49, 53.8%), had completed higher or postgraduate education (n=71, 78.0%), and had Fitzpatrick skin phototype III or IV (n=81, 89.0%). All lesions were located on the lower limbs, most frequently on the lateral (n=29, 31.9%) and medial (n=18, 19.8%) thigh. Hyperpigmentation had been present for up to six months in 89 patients (97.8%) and most commonly followed foam sclerotherapy (n=44, 48.4%). Every participant reported some degree of impact on self-esteem, rated as moderate in 35 (38.5%) and high in 46 (50.5%). Clothing was intentionally used to conceal the affected area at least occasionally by 86 patients (94.5%) and frequently or always by 56 (61.5%). Among the 89 patients whose hyperpigmentation followed varicose vein treatment, 72 (80.9%) considered the hyperpigmentation aesthetically worse than the varicose veins themselves. Most patients (n=79, 86.8%) expected at least 75% lightening of the lesions with the current treatment.

Conclusions: In this case series, impaired self-esteem, concealment behaviors, a greater aesthetic burden attributed to hyperpigmentation than to the underlying venous disease, and high treatment expectations were consistently reported. Taken together, these findings highlight the relevance of residual hyperpigmentation to the patient’s subjective experience and support the systematic use of patient-reported outcomes in the planning and evaluation of the outcomes of venous procedures. The findings should be interpreted in light of the retrospective design, single private-center setting, the characteristics of the sample, and the use of a questionnaire without psychometric validation.

## Introduction

Cutaneous hyperpigmentation is defined as a localized increase in melanin or hemosiderin in the skin, presenting as patches of varying shades that may affect exposed areas such as the legs [1]. It may arise as post-inflammatory hyperpigmentation, in which cutaneous injury and inflammation increase melanocyte stimulation (as after sclerotherapy, surgery, or trauma), or from increased hemosiderin deposition, generally in association with bruising, as may occur after surgery or sclerotherapy [1-3]. 

This complication is among the most frequent adverse events after vascular procedures, particularly sclerotherapy. Reported incidence varies across studies but is approximately 30% [2,3]. Predisposing conditions can often be identified during history taking and physical examination, including melasma, a family history of pigmentary disorders, a higher Fitzpatrick skin phototype, cumulative sun exposure, and the use of certain photosensitizing or hormonal medications. These factors may act independently or synergistically to increase the risk of hyperpigmentation [4].

Beyond its strictly clinical dimension, this condition is also relevant to psychosocial health. Although hyperpigmentation is considered a benign dermatologic condition, its visible nature often extends beyond the physical domain, causing aesthetic distress and negatively affecting an individual’s self-image [1,5]. Because the skin is exposed, it plays a central role in nonverbal communication and in the development of self-esteem; visible changes can therefore influence perceived attractiveness and self-confidence [6].

Qualitative studies have shown that patients with other forms of hyperpigmentation, such as melasma, report reduced self-esteem and quality of life associated with pigmented lesions, including feelings of shame before treatment and significant improvement after effective therapeutic intervention [5]. Similarly, studies of post-inflammatory hyperpigmentation suggest that this condition negatively affects patients’ quality of life across emotional and social domains, largely independently of the clinical severity assessed by healthcare professionals [7].

In the setting of cutaneous complications associated with venous procedures, demand for treatment of hyperpigmentation has been increasing, reflecting not only a desire for aesthetic improvement but also the pursuit of emotional well-being. Satisfaction with treatment outcomes has been identified as a factor that may positively reinforce self-esteem, with most individuals reporting increased self-confidence after therapeutic intervention [8].

Despite the high clinical prevalence of cutaneous hyperpigmentation in patients with venous disease and after the procedures used to treat it, studies of its psychosocial impact remain scarce, particularly with regard to self-esteem. Accordingly, this study aimed to describe aspects related to the perceived impact of hyperpigmentation on self-esteem in patients with cutaneous hyperpigmentation following venous procedures.

## Materials and methods

This was an observational, retrospective, descriptive case series of 91 patients with cutaneous hyperpigmentation following venous procedures who were seen between 2019 and 2025 at Seabra Excelencia Vascular, a specialized private clinic located in Vitória, Espírito Santo, Brazil. The study was approved by the Research Ethics Committee of Escola Superior de Ciências da Santa Casa de Misericórdia de Vitória (Emescam) (approval number: CAAE 93132425.7.0000.5065).

Eligibility criteria

Uniform diagnostic criteria were applied by the same clinical team throughout the study period. The medical records of the patients seen during the study period were reviewed, and those with a clinical diagnosis of cutaneous hyperpigmentation following venous procedures were considered eligible. Patients with cutaneous hyperpigmentation of non-vascular etiology, and patients with venous disease who had not undergone venous procedures, were considered ineligible.

Data collection

Data were collected with a structured questionnaire (see Appendices) routinely used in clinical practice and filed in the patients’ medical records. The instrument was developed by the medical team of the clinic on the basis of their experience with cutaneous hyperpigmentation following venous procedures; it was neither derived from nor adapted from any previously published instrument, and its purpose was to systematize data collection. It comprised closed-ended questions addressing sociodemographic and clinical characteristics, as well as self-reported items related to hyperpigmentation, including the perceived impact on self-esteem, its aesthetic repercussion, its influence on clothing choices, the history of previous treatments, and expectations regarding the current treatment. To assess the perceived impact of hyperpigmentation on self-esteem, participants answered the question “How much do these spots interfere with your self-esteem?”, rating the interference as none, mild, moderate, or high.

The questionnaire was completed in a standardized manner at the start of hyperpigmentation treatment; part of the information was provided by the patients, who answered the sociodemographic and subjective questions, and part was recorded by the attending physicians, who completed the clinical data. The instrument did not undergo formal validation or reliability testing. 

For the present study, both the data recorded in the questionnaire and the clinical information available in the medical records were used. 

Skin phototype was determined by the attending physicians during patient assessment according to the Fitzpatrick classification, a system widely used to categorize skin according to its response to sun exposure and its tendency to tan. The classification ranges from I (very fair skin, always burns, never tans) to VI (deeply pigmented skin, rarely burns) [9].

Variables and analysis

The variables analyzed included the sociodemographic characteristics of the patients, subjective aspects related to the perception and aesthetic impact of hyperpigmentation, including its perceived impact on self-esteem, and clinical data related to cutaneous hyperpigmentation. Data were organized and analyzed descriptively and are presented as absolute and relative frequencies. IBM SPSS Statistics for Windows, version 30.0 (IBM Corp., Armonk, New York, United States) was used for data analysis.

## Results

The sample consisted predominantly of women, most of whom were aged 30-49 years, married, had completed higher or postgraduate education, and had Fitzpatrick skin phototype III or IV (Table 1). All patients had hyperpigmentation on the lower limbs, most frequently on the lateral and medial thigh. The lesions were mainly associated with varicose vein treatment; foam sclerotherapy was the most frequent preceding procedure, followed by conventional surgery and liquid sclerotherapy (Table 1). At the start of hyperpigmentation treatment, 89 of the 91 patients (97.8%) had had the lesions for up to six months.

**Table 1:** Individual and hyperpigmentation-related characteristics of the patients (N = 91)

Variable	Category	Frequency	Percentage
Sex	Male	6	6.6
	Female	85	93.4
Age group (years)	20–29	9	9.9
	30–39	21	23.1
	40–49	28	30.8
	50–59	17	18.7
	≥60	16	17.6
Marital status	Single	12	13.2
	Married	59	64.8
	Stable union (common-law partnership)	6	6.6
	Divorced	4	4.4
	Widowed	10	11.0
Educational attainment	Completed primary education	3	3.3
	Completed secondary education	17	18.7
	Completed higher education	48	52.7
	Completed postgraduate education	23	25.3
Fitzpatrick skin phototype	I	1	1.1
	II	5	5.5
	III	40	44.0
	IV	41	45.1
	V	4	4.4
Main site of hyperpigmentation	Anterior thigh	9	9.9
	Posterior thigh	9	9.9
	Lateral thigh	29	31.9
	Medial thigh	18	19.8
	Anterior lower leg	6	6.6
	Posterior lower leg	4	4.4
	Lateral lower leg	4	4.4
	Medial lower leg	5	5.5
	Popliteal fossa	7	7.7
Procedure preceding the onset of hyperpigmentation	Liquid sclerotherapy	19	20.9
	Foam sclerotherapy	44	48.4
	Transdermal laser	2	2.2
	Cryo-laser and cryo-sclerotherapy (CLaCS)	5	5.5
	Conventional (open) varicose vein surgery	20	22.0
	Endovenous laser ablation	1	1.1
Duration of hyperpigmentation	≤1 month	40	44.0
	>1–3 months	34	37.4
	>3–6 months	15	16.5
	>6–12 months	2	2.2

All participants reported some degree of impact of hyperpigmentation on self-esteem, rated as moderate or high in 81 patients (89.0%) (Table 2). The intentional use of clothing to conceal the hyperpigmented area was reported at least occasionally by 86 of the 91 patients (94.5%), and frequently or always by 56 patients (61.5%). Medically indicated compression garments were not considered concealment clothing. Among the 89 patients whose hyperpigmentation followed varicose vein treatment, 72 (80.9%) considered the hyperpigmentation aesthetically more detrimental than the treated varicose veins themselves. Seventy patients (76.9%) had previously attempted treatments to lighten the lesions, predominantly without significant improvement. Despite this, 79 patients (86.8%) expected at least 75% lightening of the lesions with the current treatment (Table 2).

**Table 2:** Perceived impact of hyperpigmentation on self-esteem and related variables ᵃ Two patients whose previous venous procedure had not been performed for varicose vein treatment were excluded from this variable. Because the denominator differs across variables, no overall total row is presented.

Variable	Category	Frequency	Percentage
Perceived impact of hyperpigmentation on self-esteem (N = 91)	None	0	0.0
	Mild	10	11.0
	Moderate	35	38.5
	High	46	50.5
Use of clothing to conceal the hyperpigmentation (N = 91)	Never	5	5.5
	Rarely	7	7.7
	Sometimes	23	25.3
	Frequently	40	44.0
	Always	16	17.6
Comparative aesthetic assessment: varicose veins vs. hyperpigmentation (n = 89)ᵃ	Varicose veins worse	3	3.4
	Equivalent	14	15.7
	Hyperpigmentation worse	72	80.9
Previous attempt at treatment (N = 91)	No	21	23.1
	Yes, with no improvement	47	51.6
	Yes, with slight improvement	23	25.3
Expected lightening with the current treatment (N = 91)	100% of the hyperpigmented area	11	12.1
	75–99%	68	74.7
	50–74%	11	12.1
	25–49%	1	1.1

In the descriptive analysis by sex, a moderate-to-high impact on self-esteem was reported by 76 of 85 women (89.4%) and by five of the six men (83.3%). Clothing was used frequently or always by 54 of 85 women (63.5%) and by two of the six men (33.3%). Given that only six men were included, these sex-stratified proportions are exploratory and should be interpreted with caution. Also in the descriptive analysis, some degree of impact on self-esteem was reported in every age group, with the proportion rating the impact as high being greatest among patients aged 60 years or older (10/16, 62.5%) (Table 3).

**Table 3:** Perceived impact of hyperpigmentation on self-esteem, by age group

Age group (years)	None, n (%)	Mild, n (%)	Moderate, n (%)	High, n (%)	Total, n
20–29	0 (0.0)	1 (11.1)	5 (55.6)	3 (33.3)	9
30–39	0 (0.0)	1 (4.8)	10 (47.6)	10 (47.6)	21
40–49	0 (0.0)	4 (14.3)	8 (28.6)	16 (57.1)	28
50–59	0 (0.0)	2 (11.8)	8 (47.1)	7 (41.2)	17
≥60	0 (0.0)	2 (12.5)	4 (25.0)	10 (62.5)	16
All patients	0 (0.0)	10 (11.0)	35 (38.5)	46 (50.5)	91

## Discussion

The results of the present study suggest that cutaneous hyperpigmentation following venous procedures carries a substantial perceived impact on patients’ self-esteem, predominantly rated as moderate or high, and is accompanied by concealment behaviors. These findings are consistent with literature showing that visible pigmentary changes, even when clinically benign, are associated with meaningful impairment of self-image and emotional well-being [10,11].

The skin plays a central role in the construction of bodily identity and functions as an important element of nonverbal communication. Evidence suggests that persistent changes in skin color, such as hyperpigmentation following venous procedures, may acquire a subjective significance disproportionate to their pathophysiological severity, particularly because they are visible and difficult to conceal [12]. Hyperpigmentation may thus be perceived as a marker of aesthetic imperfection, negatively affecting self-confidence and satisfaction with one’s own body image. This may help explain the frequent reports of perceived impact on self-esteem and the concealment behaviors observed in this case series [13].

In the context of elective procedures, the discrepancy between conventional medical assessment, which tends to classify hyperpigmentation as a minor adverse event after venous procedures, and the patient’s subjective experience underscores the importance of incorporating patient-reported outcomes into clinical practice. Studies have shown that a patient’s perception of their skin condition is an important determinant of self-esteem, regardless of objective clinical severity [14,15].

The habitual use of clothing to conceal the lesions observed in this study suggests the adoption of coping strategies in response to aesthetic distress. Similar findings have been described in individuals with visible dermatoses, in whom concealment behaviors are frequently associated with feelings of shame and social avoidance [16,17]. Such factors may substantially affect quality of life and may weigh on the decision of whether to undergo a vascular procedure [18].

The higher frequency of concealment behaviors observed among women is consistent with evidence that visible skin changes may carry greater emotional and psychosocial repercussions in this group. Studies indicate that changes in skin appearance can impair self-esteem and intensify feelings of shame, social anxiety, and isolation, particularly in sociocultural contexts in which women’s appearance is subject to more rigid aesthetic standards. This may favor avoidance behaviors and social withdrawal, not only as an individual response to the skin condition but also as a reflection of broader social processes that link personal worth to physical appearance [19-22]. Among men, the lower frequency of concealment behaviors observed here may represent a distinct way of experiencing the condition, shaped by social norms and gender-related behavioral patterns [23-25]. Because only six men were included, however, this comparison is hypothesis-generating rather than confirmatory.

Reports of a perceived impact on self-esteem were frequent across all age groups, including among individuals aged 60 years or older. This is consistent with studies showing that concern with appearance and distress associated with visible changes persist throughout the life course, although their intensity may vary between age groups [26,27].

With regard to treatment expectations, the results show a predominance of high expectations for lesion lightening, with a substantial proportion of participants seeking improvement of at least 75%. This pattern suggests that pigmentary changes may become central to the patient’s illness experience, often linked to psychosocial repercussions that extend beyond the objective clinical severity of the condition. The phenomenon is recurrent in therapeutic contexts in which aesthetic impairment plays a central role in the perception of illness and in the evaluation of treatment outcomes [28,29]. Conversely, although the proportion of individuals with more conservative expectations was small, this may reflect previous frustrating treatment experiences, which tend to lower expectations regarding subsequent treatments [30,31].

Previous studies indicate that gaps in physician-patient communication about therapeutic limitations contribute to persistent emotional distress and to reduced overall satisfaction with care [32]. Prior alignment of expectations therefore appears to be an integral component of the clinical management of hyperpigmentation.

The perception of a greater aesthetic burden attributed to hyperpigmentation than to the varicose veins themselves suggests that persistent cutaneous sequelae may be experienced as more troubling than the treated vascular disease. This phenomenon has been described in the literature, in which visible residual marks may be interpreted as treatment failure even when the primary clinical objective has been achieved [33,34].

Therapeutic success should therefore be evaluated comprehensively, considering not only anatomical and functional resolution but also the factors that shape the patient’s aesthetic and emotional perceptions. Treatment generally aims to improve the appearance of the limb; the development of hyperpigmentation after the intervention runs counter to that aim and can generate dissatisfaction [35,36].

From a clinical standpoint, these findings reinforce the need for a broader approach to vascular care, one that recognizes cutaneous hyperpigmentation not merely as a secondary aesthetic consequence but as a condition potentially associated with psychosocial distress [37]. The high frequency of self-reported impact on self-esteem and of concealment behaviors observed in this series indicates that patient-perceived outcomes should be considered systematically when therapeutic results are evaluated. Incorporating patient-reported outcome measures (PROMs) into assessment may contribute to more person-centered care by facilitating expectation alignment and more informed clinical decision-making [38].

Limitations

This study has several limitations. First, its retrospective design and the inclusion of patients from a single private clinic introduce potential selection bias and limit generalizability. The sample was predominantly female and had a high educational level, characteristics that, together with the fact that these patients sought treatment for hyperpigmentation at a private clinic, may have favored the inclusion of individuals with a greater perception of the impact of this complication, potentially resulting in an overestimation of that impact. Therefore, the results may not be representative of all patients who develop hyperpigmentation after venous procedures, particularly those with different sociodemographic characteristics, those who do not seek treatment for this complication, or those treated in other care settings. Second, the instrument used to assess the perceived impact of hyperpigmentation on self-esteem has not undergone psychometric validation, which limits comparability with the literature, hinders standardization of outcome measurement, and introduces potential information bias. Third, almost the entire sample had lesions of up to six months’ duration, which precluded any analysis of how the persistence of hyperpigmentation over time affects its perceived impact and restricts extrapolation to patients with longer-standing lesions. Fourth, the small number of male participants (n = 6) means that the sex-stratified findings are exploratory. Finally, no inferential analysis was performed, so associations between variables could not be statistically assessed. In this context, the results should be interpreted with caution and confirmed in prospective studies using validated instruments and including patients at different stages of the condition [15].

## Conclusions

In this case series, patients frequently reported a perceived impact of hyperpigmentation on self-esteem, adopted strategies to conceal the lesions, attributed a greater aesthetic burden to hyperpigmentation than to the condition that prompted the procedure, and expressed high expectations regarding the treatment of this complication. Taken together, these findings highlight the relevance of residual hyperpigmentation to the patient’s subjective experience. Despite the study’s limitations, these findings reinforce the importance of considering the patient’s perspective in the planning and evaluation of the outcomes of venous procedures.

## Figures and Tables

**Figure 1:**
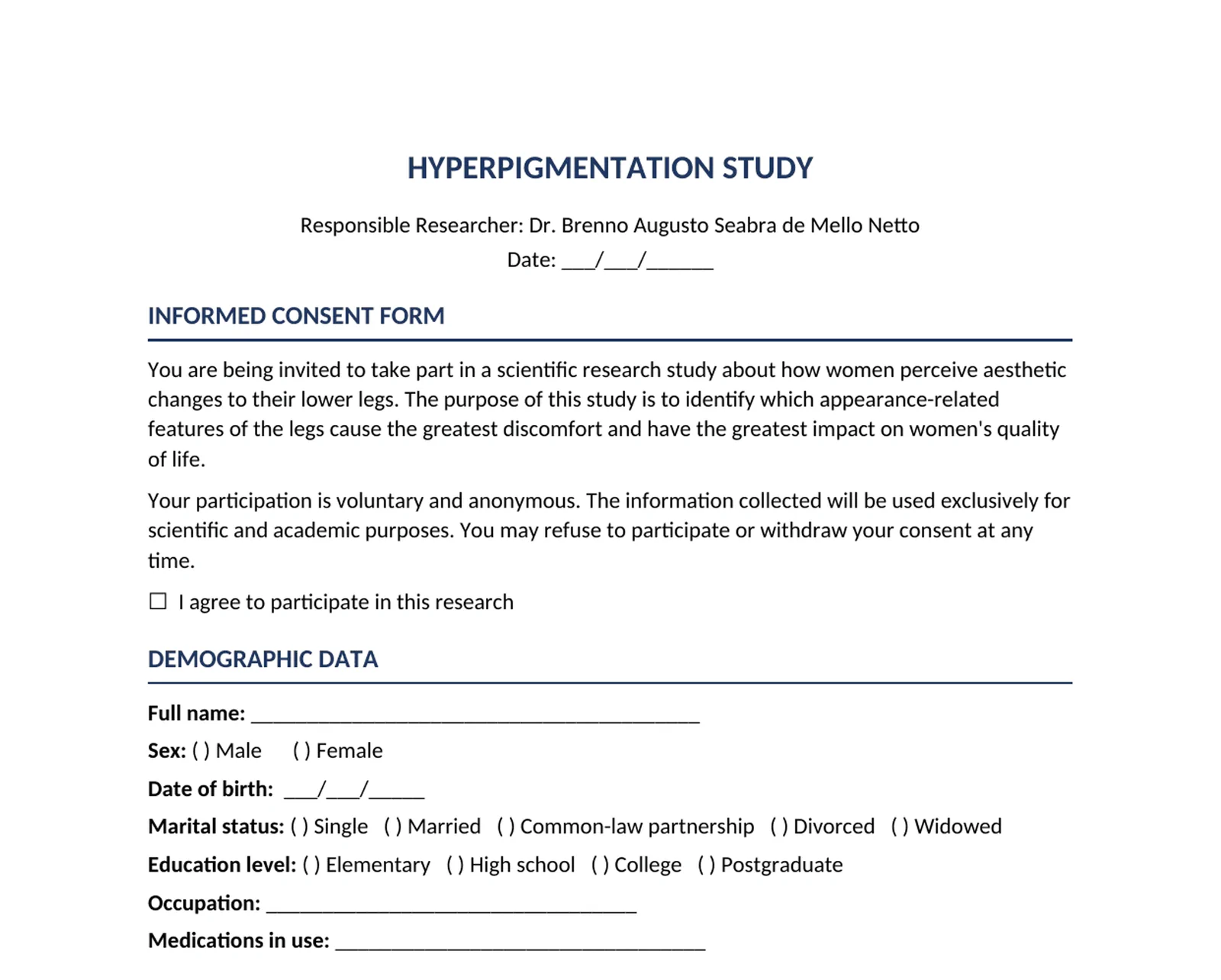
Study questionnaire, page 1

**Figure 2:**
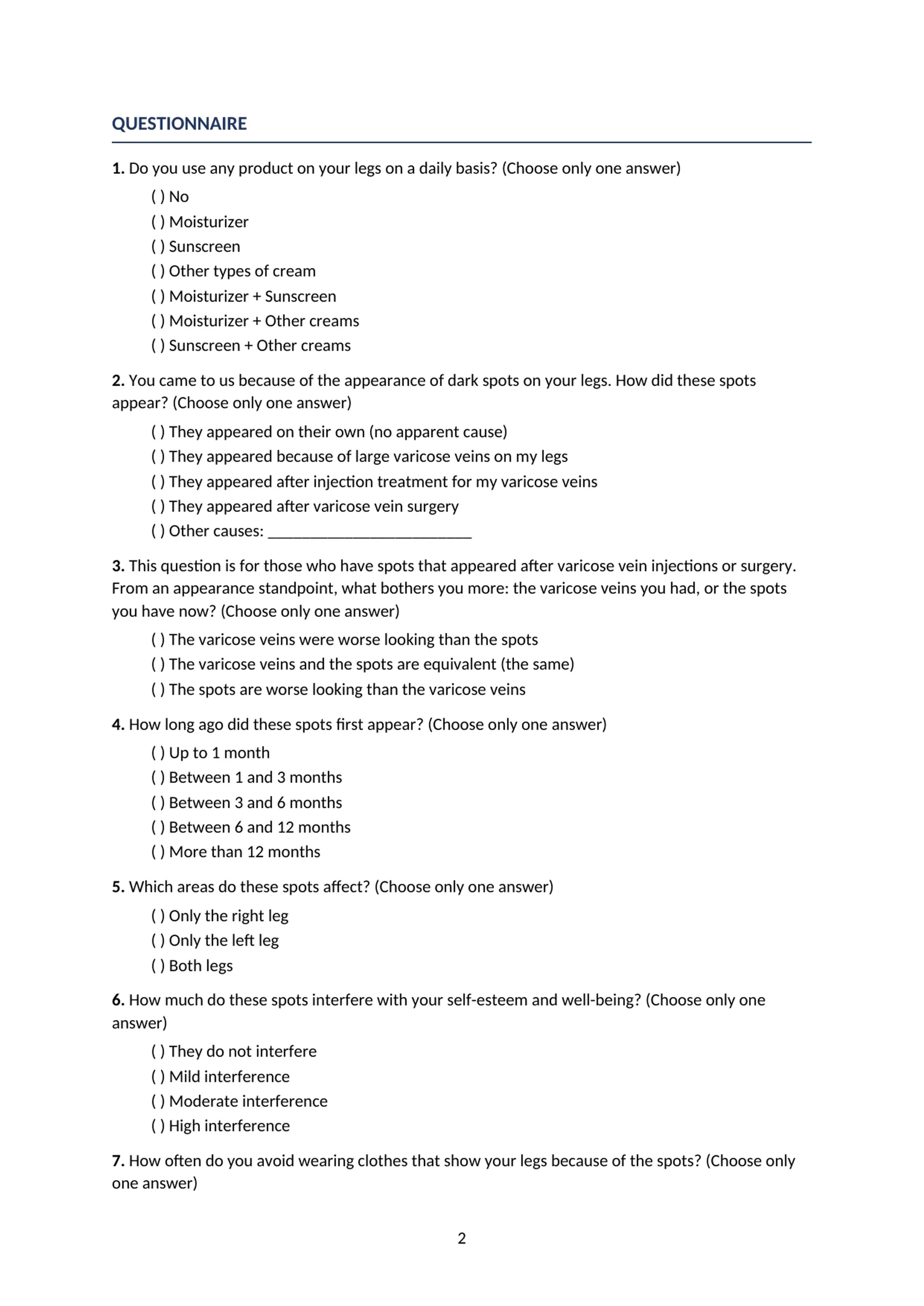
Study questionnaire, Page 2

**Figure 3:**
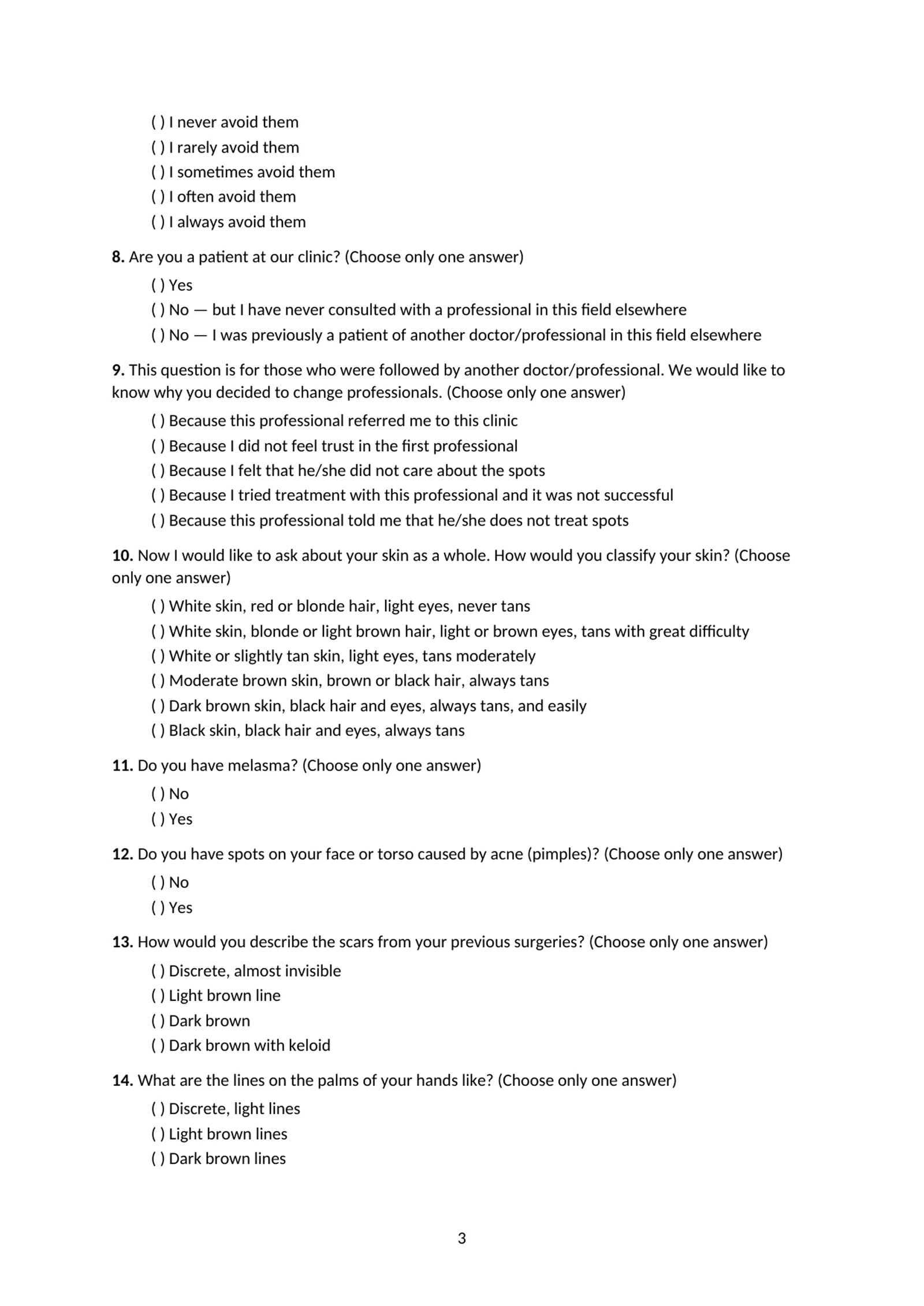
Study questionnaire, Page 3

**Figure 4:**
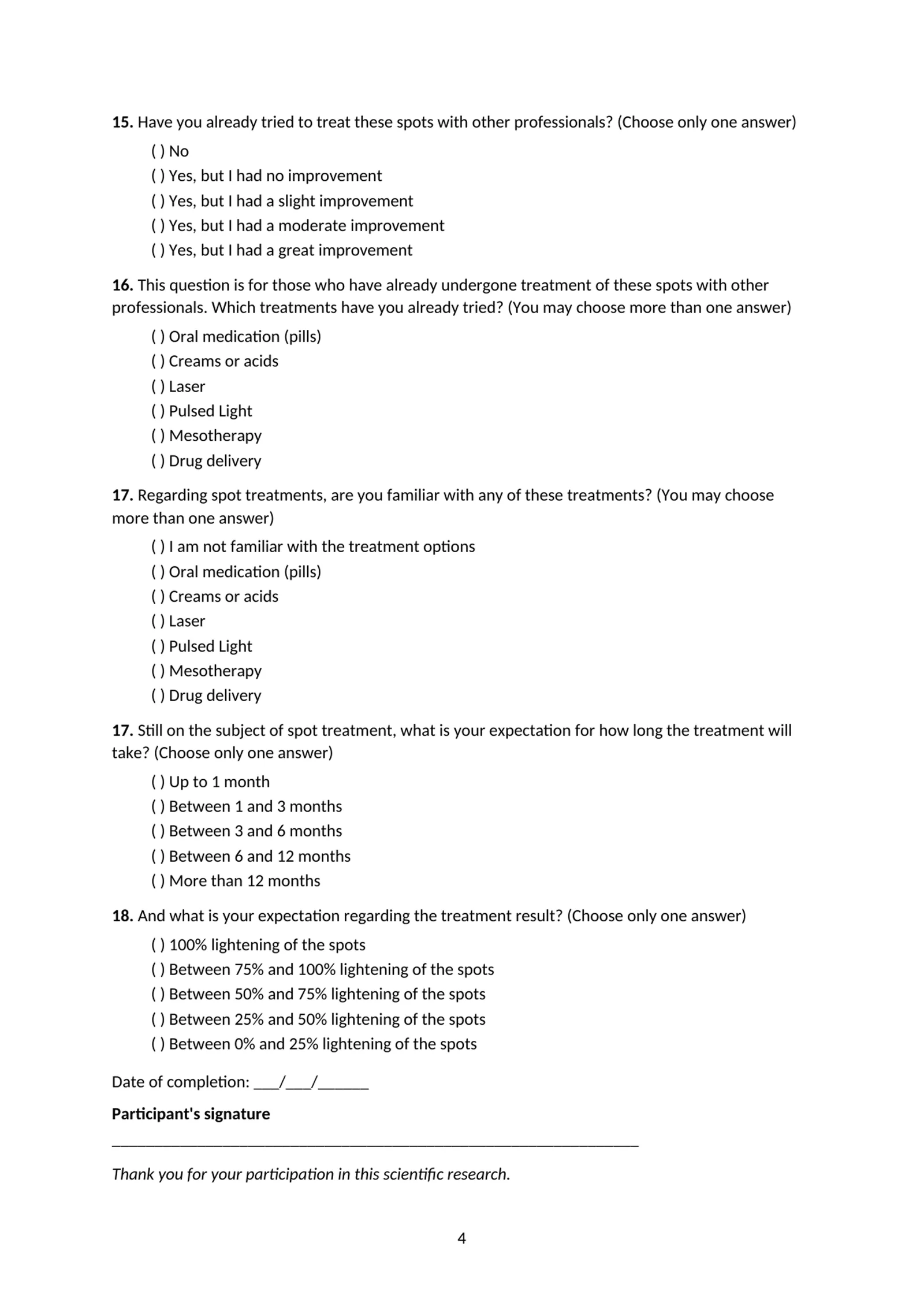
Study questionnaire, Page 4

**Figure 5:**
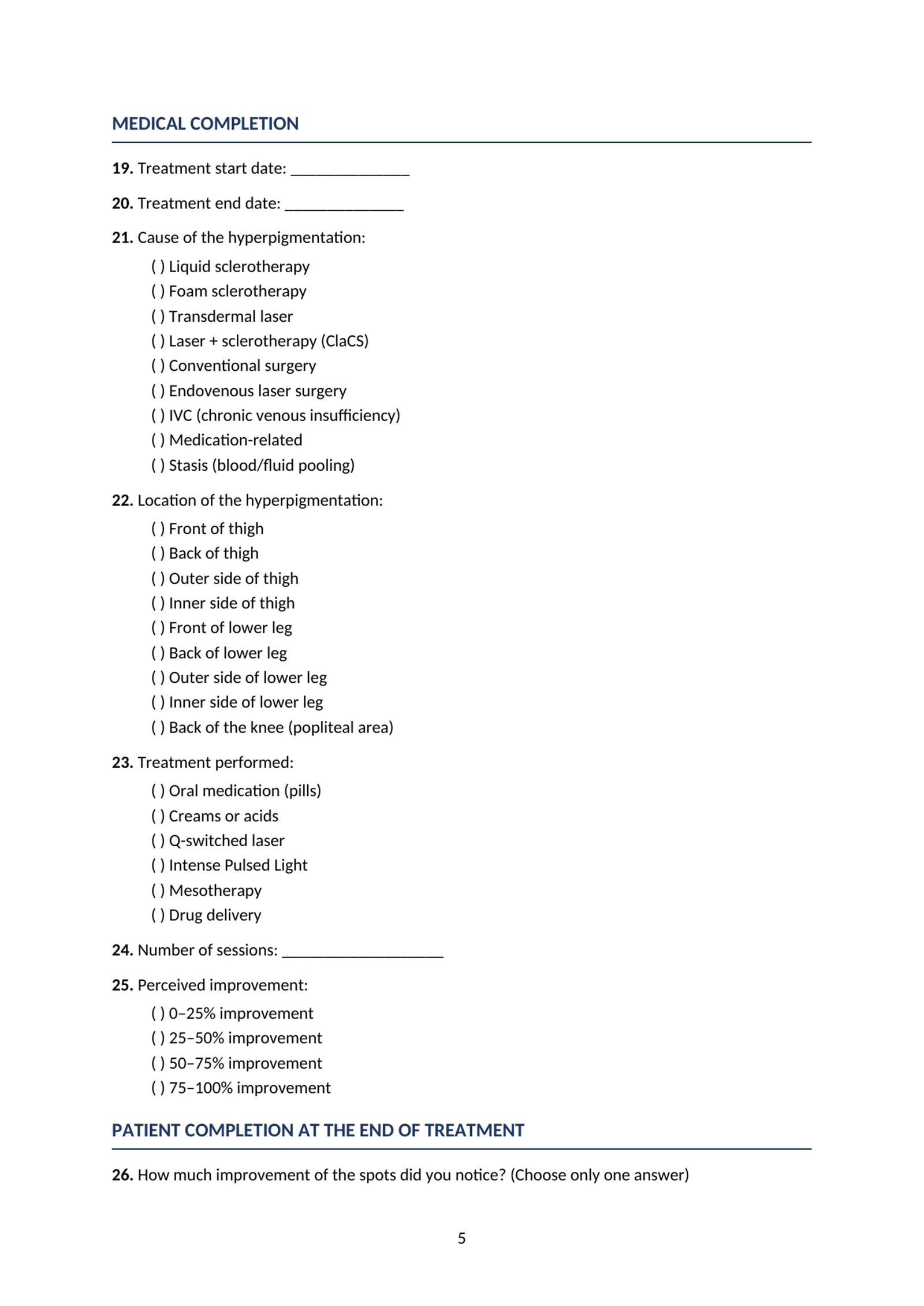
Study questionnaire, Page 5

**Figure 6:**
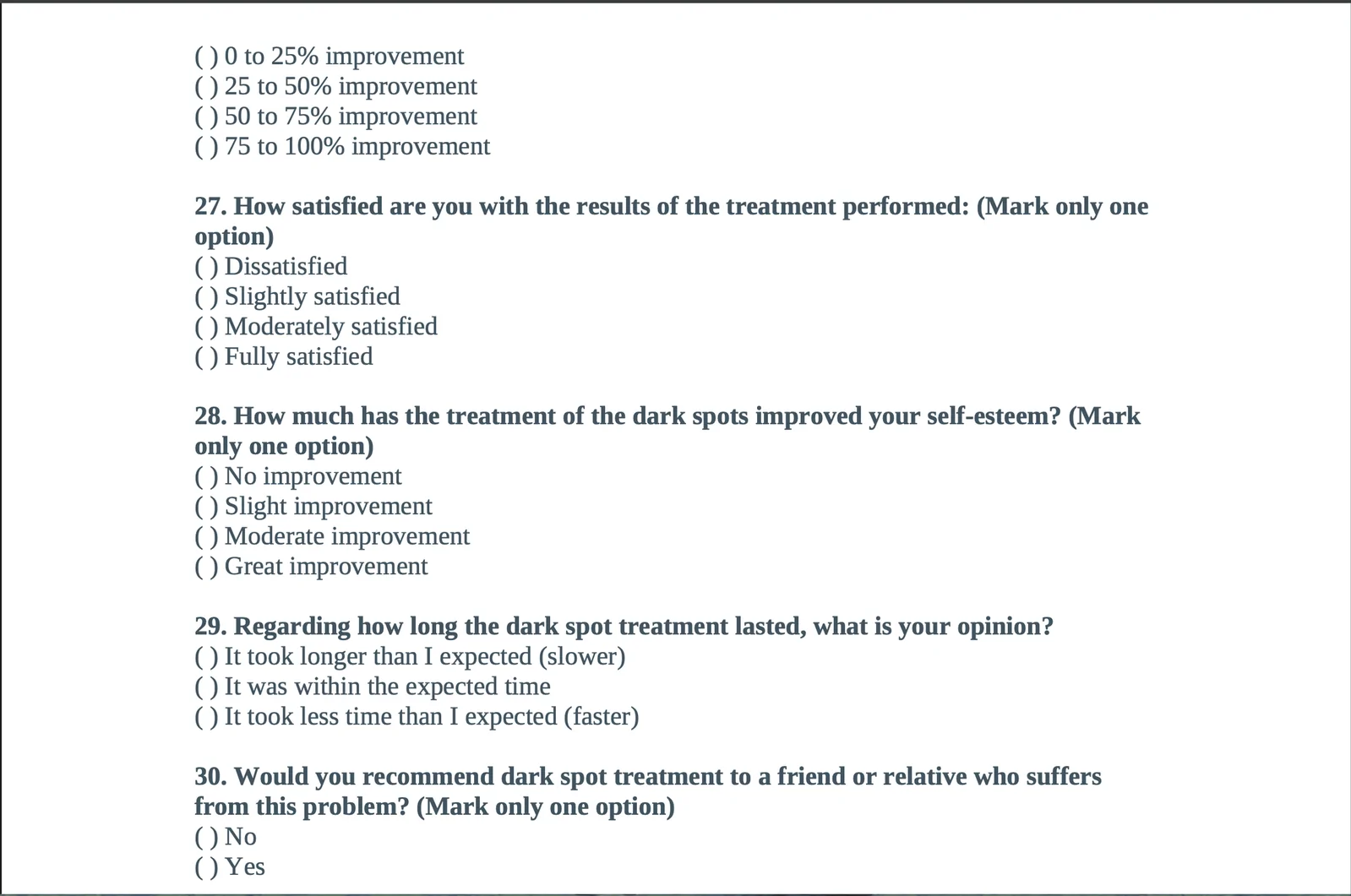
Study questionnaire, Page 6
